# Supplementary material for: Isolated abnormal diffusing capacity for carbon monoxide (iso↓DLco) is associated with increased respiratory symptom burden in people with HIV infection
Source: PLoS One. 2023 Jul 18;18(7):e0288803. doi: 10.1371/journal.pone.0288803 (PMC10353811; doi:10.1371/journal.pone.0288803)
Supplement: S1 Table — (DOCX) [file pone.0288803.s002.docx]

**S1 Table: Outcomes of multivariable analyses of predictors associated with abnormal DLco.**

| mMRC | OR | 95% CI | p-value |
| --- | --- | --- | --- |
| Location | 0.067 | 0.010, 0.458 | **0.006** |
| BMI | 1.136 | 1.059,1.219 | **<0.001** |
| Male sex | 1.421 | 0.397, 5.091 | 0.589 |
| Ever smoker | 1.697 | 0.708, 4.066 | 0.236 |
| Age (years) | 1.023 | 0.990, 1.057 | 0.178 |
| Bacterial pneumonia ever | 3.786 | 1.607, 8.919 | **0.002** |

**S1a Table: Additional predictors associated with abnormal DLco in mMRC multivariable logistic regression model.**

| CAT | β | 95% CI | p-value |
| --- | --- | --- | --- |
| BMI | 0.406 | 0.223, 0.590 | **<0.001** |
| Male sex | 0.209 | -3.209, 3.627 | 0.905 |
| Ever smoker | 3.292 | 0.928, 5.655 | **0.006** |
| Age (years) | -0.058 | -0.165, 0.050 | 0.295 |
| Bacterial pneumonia ever | 4.746 | 2.351, 7.141 | **<0.001** |

**S1b Table: Additional predictors associated with abnormal DLco in CAT multivariable linear regression model.**

| SGRQ | β | 95% CI | p-value |
| --- | --- | --- | --- |
| Location | -13.650 | -17.927, -9.373 | **<0.001** |
| BMI | 1.028 | 0.522, 1.534 | **<0.001** |
| Male sex | -4.240 | -12.757, 4.278 | 0.329 |
| Ever smoker | 6.660 | 1.109, 12.210 | **0.019** |
| Age (years) | 0.003 | -0.220, 0.226 | 0.976 |
| Bacterial pneumonia ever | 8.939 | 3.349, 14.529 | **0.002** |
| Ever injection drug use | 7.419 | 1.230,13.607 | **0.019** |

**S1c Table: Additional predictors associated with abnormal DLco in SGRQ multivariable linear regression model.**
